# Perirenal fat thickness as a superior obesity-related marker of subclinical carotid atherosclerosis in type 2 diabetes mellitus

**DOI:** 10.3389/fendo.2023.1276789

**Published:** 2023-10-27

**Authors:** Xiu Li Guo, Jian Wen Wang, Mei Tu, Wei Wang

**Affiliations:** Longyan First Affiliated Hospital of Fujian Medical University, Longyan, Fujian, China

**Keywords:** obesity, perirenal fat thickness, sub-clinical carotid atherosclerosis, carotid intima-to-media thickness, type 2 diabetes mellitus

## Abstract

**Objective:**

Emerging evidence highlighted that perirenal adipose tissue might regulate the cardiovascular and metabolism system through several pathways. This study aimed to assess the association between perirenal fat thickness (PrFT) and subclinical carotid atherosclerosis (SCCA) in type 2 diabetes mellitus (T2DM).

**Method:**

A total of 670 participants with complete data were included in this study. The trained reviewer collected demographic and anthropometric information. Laboratory assessments were determined by standard methods. PrFT and SCCA were evaluated by computed tomography and ultrasound. Binomial logistic regression analysis was conducted to assess the association between PrFT and SCCA. Receiver operating characteristic (ROC) curve analysis was conducted to evaluate the identifying value of PrFT for SCCA.

**Results:**

Overall, the prevalence of SCCA was 61.8% in T2DM. PrFT was significantly increased in the SCCA group. Growing trends were observed in the prevalence of hypertension, carotid intima-media thickness (cIMT) > 1, plaque, and SCCA across the PrFT quartiles. Spearman correlation analysis revealed that PrFT was positively associated with cIMT (*r* = 0.401, *p* < 0.001). This correlation remained significant after adjustment for visceral fat area (VFA), subcutaneous fat area (SFA), and traditional metabolic risk factors (*β* = 0.184, *p* < 0.001). Meanwhile, PrFT was independently correlated with plaque, cIMT > 1 mm, and SCCA. The ORs (95% CI) were 1.072 (1.014–1.135), 1.319 (1.195–1.455), and 1.216 (1.119–1.322). Furthermore, PrFT remained correlated considerably with SCCA in subgroup analysis after stratification for age, sex, smoking, hypertension, and body mass index. From the ROC curve analysis, the AUCs (95% CI) of PrFT, VFA, and SFA identifying SCCA were 0.794 (0.760–0.828), 0.760 (0.724–0.796), and 0.697 (0.656–0.737), respectively. The AUC of PrFT was significantly higher than VFA (*p* = 0.028) and SFA (*p* < 0.001). The optimal cutoff values of PrFT were 14.0 mm, with a sensitivity of 66.7% and a specificity of 76.2%.

**Conclusion:**

PrFT was independently associated with cIMT, plaque, cIMT > 1 mm, and SCCA as a superior obesity-related marker of SCCA in T2DM.

**Clinical trial registration:**

Clinical Trials.Gov, identifier ChiCTR2100052032.

## Introduction

Subclinical carotid atherosclerosis (SCCA) is a chronic inflammatory disease occurring in the carotid artery wall, which manifests as increased carotid intima-to-media thickness (cIMT) and plaque formation without any symptoms (1). Evidence from meta-analysis demonstrated SCCA (2). Type 2 diabetes mellitus (T2DM) is closely associated with CVD, contributing to being an independent risk factor for CVD. Despite the incidence of CVD, cardiovascular mortality in T2DM has decreased over the past decades due to the advances in CVD prevention (3, 4). The latest epidemiological surveys also revealed that CVD was the major cause of death in T2DM (5). Notably, the current practice guidelines for managing CVD in T2DM are shifting from glucose-centric strategies to a more personalized patient-centered approach. Developing effective strategies to prevent SCCA progression to CVD is essential. Identifying risk factors is the priority in developing effective prevention strategies.

Obesity is the major risk factor linked to SCCA with T2DM, and its rapidly increased prevalence drives the increase of SCCA in T2DM. Meanwhile, obesity can lead to dyslipidemia, hyperglycemia, increased oxidative stress, insulin resistance, and systemic inflammation (6), which also accelerates the development of SCCA in T2DM. Excessive accumulation of subcutaneous and visceral fat is the main manifestation of obesity. Compared to subcutaneous adipose tissue, visceral adipose tissue (VAT) plays more functional roles in the pathogenesis of CVD, metabolic syndrome (MetS), and T2DM (7, 8). Owing to more visceral fat accumulation, T2DM appeared to have more obesity-related consequences like CVD, stroke, and metabolic disorders (9). The association between VAT and SCCA has been explored in previous studies. Clinical studies observed that VAT volume and epicardial fat thickness (EFT) were independently associated with SCCA after adjustment for traditional cardiometabolic risk factors (10, 11). In addition to the conventional VAT and epicardial fat, emerging evidence highlighted that perirenal adipose tissue might play essential roles in regulating the cardiovascular system and has the potency to be a new target for CVD prevention (12).

Perirenal adipose tissue (PAT) is a kind of measurable VAT located in the retroperitoneal space. Compared with other VATs, PAT has a complete vascular supply and lymphatic system, which provide the structural basis for regulating the cardiovascular and metabolism systems (13, 14). Favre et al. found that perirenal fat thickness (PrFT) measured with computed tomography (CT) is a reliable estimate of PAT mass (15). As a marker of SCCA, several clinical studies revealed that PrFT was positively associated with cIMT in children and HIV-1-infected patients receiving highly active antiretroviral therapy (16, 17). Furthermore, the clinical study also found that the visceral fat area (VFA) had a good diagnostic value for SCCA in Japanese patients (18). Based on these findings, we can assume that PrFT may also have a good identifying value for SCCA as VFA. In addition, work completed on data showed that the association between PrFT and SCCA remained uncertain. Hence, this study aimed to explore the association between PrFT and SCCA to identify this novel risk factor for CVD. At the same time, this study would further compare the value of PrFT and VFA in identifying SCCA.

## Materials and methods

### Study population

**Figure 1:**
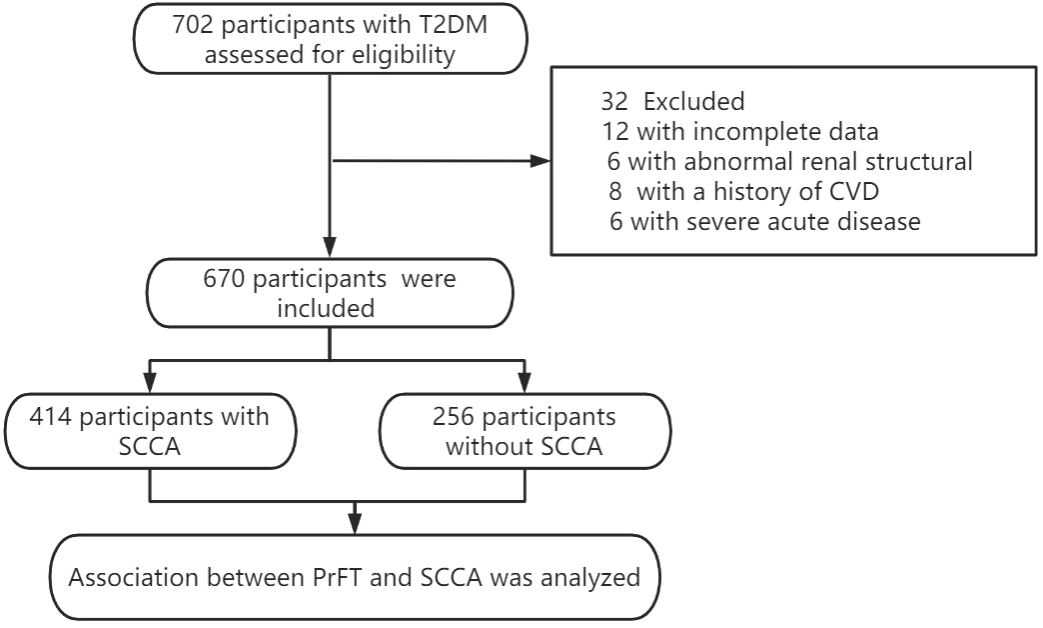
Flow diagram of the participants excluded and included in this study.

This cross-sectional study consecutively recruited T2DM admitted to the metabolic management center at Longyan First Affiliated Hospital of Fujian Medical University from December 2022 to June 2023. The study exclusion criteria were as follows: (1) with a special type of diabetes (e.g., monogenic diabetes syndromes, diseases of the exocrine pancreas, and drug- or chemical-induced diabetes), type 1 diabetes mellitus, and gestational diabetes mellitus; (2) with a history of previous CVD; (3) with renal structure abnormalities (e.g., a history of renal region surgery, presence of renal tumors and cysts, or perirenal inflammation exudates); (4) with special conditions preventing completion of CT or ultrasound examination (e.g., pregnancy, severe spinal curvature, or allergic to ultrasound coupling agents); and (5) with incomplete data. Previous studies reported that the prevalence of SCAA is approximately 50% to 60% in T2DM (19). Hence, this study planned a sample size of 600–700 T2DM according to a multiple binomial logistic regression model requirement to evaluate the association between PrFT and SCCA by SASS software statistics. Overall, this study screened 702 participants. The final analysis included 670 participants, meeting the inclusion and exclusion criteria. The flowchart of excluded and included participants is presented in **Figure 1**. This study was approved by the Ethical Committee of Longyan First Affiliated Hospital of Fujian Medical University (LY-2020069) and registered in Clinical Trials. Gov (ChiCTR2100052032). Study procedures were conducted in compliance with the Declaration of Helsinki. Informed consent was obtained from all participants.

### Clinical and laboratory assessments

The demographic data about medical history and lifestyle, including smoking, history of diseases or surgery, age, and gender, were collected by trained interviewers using a standard questionnaire and reviewing medical records. The current smoking status was defined as participants who continually smoked seven cigarettes a week for over 6 months. Anthropometric information, including height, weight, and blood pressure (BP), was measured by trained nurses using standardized methods. During height and weight measurements, participants wore hospital gowns and were barefoot. An electronic height and weight measuring device measured weight and height. Body mass index (BMI) was calculated as the weight divided by the square of height (kg/m^2^). On three occasions, an electronic sphygmomanometer measured systolic BP (SBP) and diastolic BP (DBP) with an appropriate cuff size. The mean of three readings was calculated as the final BP.

Laboratory assessments were determined by standardized methods using fasting venous blood samples. The measurement of fasting blood glucose (FBG), triglycerides (TG), total cholesterol (TC), low-density lipoprotein cholesterol (LDL-c), high-density lipoprotein (HDL-c), uric acid (UA), creatinine, and alanine aminotransferase was determined by an auto-biochemical analyzer (Roche Diagnostics Corporation). Glycosylated hemoglobin A1c (HbA1c) was measured by high-performance liquid chromatography with a D10 set (Bio-Rad). Insulin resistance was evaluated by the homeostasis model assessment (HOMA-IR): fasting serum insulin (µU/mL) × FBG (mmol/L)/22.5.

### Measurement of PrFT

Participants underwent CT scanning via Revolution VCT 128 (General Electric, Milwaukee, USA) to obtain renal structure images while in a supine position. The CT scanning area was covered between the pubic symphysis and the 10th thoracic vertebra. Experienced radiologists reconstructed the images using Advantage Windows 4.4 software (GE, Milwaukee, USA) to obtain 1.25-mm-thick consecutive slices. PrFT was measured using the method first proposed by the Mayo Clinic, which is widely used to measure PrFT. The detailed measurement was as follows (20): (1) The window center is set at −100 HU, and widths range from −50 to −200 HU. (2) PAT was differentiated from other tissues at the renal venous plane (*) by density (21). (3) The average maximal distance (blue arrow line) between the kidney’s posterior wall and the abdominal wall’s inner limit on the left and right side was measured as PrFT (**Supplementary Figure 1**). Two radiologists blinded to clinical findings were involved in measuring PrFT to reduce inter-operator variability. The inter-operator agreement between the two radiologists is 0.89. Furthermore, the average maximal distance of renal length at the coronal plane on both kidneys was measured as renal diameters.

### Measurement of VFA

Participants were performed with the abdominal bioelectrical impedance analysis method (DUALSCAN HDS-2000, Omron, Japan) to measure VFA and subcutaneous fat area (SFA) in a supine position. Experienced operators conducted the measurement according to the instrument operating manual: (1) current 1 (500 μA, 50 kHz) was applied to four pairs of electrodes fixed bilaterally between the hand and foot, and the voltage was measured axially on the abdomen; (2) a constant current (500 μA, 50 kHz) was applied to the eight electrodes placed around the trunk at the umbilical level; and (3) lean body mass was calculated by abdominal axis impedance. The mean voltage between abdominal surface electrodes calculated SFA. VFA was calculated by the two impedance data sets combined with abdominal shape parameters.

### Assessment of SCCA

Participants were performed with high-resolution B-mode carotid ultrasonography (EPIQ 5, Philips, Andover, MA) with an L12-5 MHz ultrasonic probe to measure the carotid arteries (common carotid artery, internal carotid artery, and external carotid artery) according to the standardized protocol (21). Participants were examined supine with slight neck hyper-extension towards the contralateral side. Common carotid arteries with the regular lumen–intima interface parallel to the adventitia on both sides were captured for further measurements. The average intima-media thickness (IMT) of the far vessel wall at a site approximately 1 cm proximal to the carotid bulb was calculated as cIMT. Carotid plaque was defined as a focal structure that protruded at least 0.5 mm into the arterial lumen, vascular lumen thickness greater than 50% of the surrounding IMT, or IMT > 1.5 mm. All operations were performed by experienced ultrasonologists who were blind to clinical information. The inter-operator agreement between the two ultrasonologists is 0.93. SCCA was defined as participants with cIMT > 1.0 mm or (and) carotid plaque (22).

### Statistical analysis

Statistical analysis was performed using SPSS 23.0 software (SPSS Inc. IBM). Descriptive data are expressed as means ± standard deviation (SD). Discrete variables were summarized in frequency tables (*N*, %). The difference in baseline characteristics between SCCA and non-SCCA groups was analyzed by the independent samples *t*-test or Kruskal–Wallis test. Furthermore, a one-way analysis of variance followed by the Tukey test for multiple comparisons was conducted to investigate the difference in metabolic risk factors among PrFT quartiles. The chi-squared (*χ* ^2^) test or Fisher exact test was used to compare categorical variables. The relationship between PrFT and cIMT was assessed using Spearman correlation analysis. Multiple regression analysis was used to estimate the independent correlation of PrFT with cIMT. Binomial logistic regression analysis was used to estimate the independent variable of PrFT for SCCA. The receiver operating characteristic curves were used to compare the identifying value of SCCA between PrFT and VFA. A two-tailed value of *p* < 0.05 was considered statistically significant.

## Results

### Baseline characteristics of the study population

Overall, the mean age of participants was 53.1 ± 8.3 years old, with a mean diabetic duration of 5.1 ± 3.2 years. The mean PrFT was 12.7 ± 4.7 mm. **Table 1** summarizes the baseline characteristics of participants in the SCCA and non-SCCA groups. In the SCCA group, the cardiometabolic risk factors like age, BMI, WC, SBP, DBP, TG, TC, LDL-c, UA, HOMA-IR, SFA, and VFA were statistically higher than the non-SCCA group (*p* < 0.05). Meanwhile, the SCCA group was more hypertensive and smoked more than the non-SCCA group (*p* < 0.05). In contrast, the HDL-c was decreased in the SCCA group (*p* < 0.05). As expected, the PrFT was also increased in the SCCA group compared with the non-SCCA group (12.7 ± 4.7 mm vs. 9.7 ± 4.2 mm, *p* < 0.05).

**Table 1:** Baseline characteristics of participants in SCCA and non-SCCA groups.

Variable	Total (*n* = 670)	SCCA (*n* = 414)	Non-SCCA (*n* = 256)	*p*
Age (years)	53.1 ± 8.3	54.9 ± 8.4	50.1 ± 7.2	<0.001
Men, *n* (%)	352 (52.5)	217 (52.4)	135 (52.7)	0.488
Duration (years)	5.1 ± 3.2	5.5 ± 3.2	4.5 ± 3.1	<0.001
BMI (kg/m^2^)	24.5 ± 2.9	25.4 ± 2.9	23.0 ± 2.1	<0.001
WC (cm)	85.8 ± 6.9	87.8 ± 7.2	82.3 ± 4.8	<0.001
SBP (mmHg)	133.7 ± 17.6	139.5 ± 17.2	124.5 ± 14.1	<0.001
DBP (mmHg)	81.7 ± 9.7	83.5 ± 8.6	78.2 ± 10.4	<0.001
HbA1c (%)	8.7 ± 1.1	8.8 ± 1.2	8.6 ± 0.9	0.071
TG (mmol/L)	2.14 ± 1.33	2.58 ± 1.45	1.44 ± 0.68	<0.001
TC (mmol/L)	5.17 ± 1.20	5.27 ± 1.23	5.02 ± 1.02	<0.001
HDL-c (mmol/L)	1.10 ± 0.25	1.04 ± 0.24	1.20 ± 0.22	<0.001
LDL-c (mmol/L)	3.55 ± 0.96	3.59 ± 0.99	3.41 ± 0.90	0.048
UA (μmol/L)	364.5 ± 85.3	383.7 ± 87.6	333.5 ± 75.1	<0.001
Creatinine (μmol/L)	70.3 ± 13.1	70.3 ± 12.9	70.4 ± 13.6	0.924
ALT (IU/L)	33.9 ± 8.7	34.7 ± 8.8	33.4 ± 8.6	0.067
HOMA-IR	3.19 ± 1.74	6.27 ± 3.02	4.38 ± 2.72	<0.001
PrFT (mm)	12.7 ± 4.8	14.6 ± 4.0	9.7 ± 4.2	<0.001
VFA (cm^2^)	89.1 ± 17.3	91.6 ± 17.1	85.2 ± 14.7	<0.001
SFA (cm^2^)	167.2 ± 27.7	171.3 ± 27.6	160.5 ± 25.9	<0.001
Renal diameter (cm)	11.4 ± 1.6	11.4 ± 1.5	11.3 ± 1.8	0.862
Hypertension, *n* (%)	244 (36.0)	205 (49.5)	39 (15.2)	<0.001
Smoking, *n* (%)	256 (38.2)	182 (44.0)	69 (27.0)	<0.001

BMI, body mass index; WC, waist circumference; SBP, systolic blood pressure; DBP, diastolic blood pressure; HbA1c, glycated hemoglobin A1c; TG, triglyceride; TC, total cholesterol; HDL-c, high-density lipoprotein cholesterol; LDL-c, low-density lipoprotein cholesterol; UA, uric acid; ALT, alanine aminotransferase; HOMA-IR, homeostasis model assessment insulin resistance; VFA, visceral fat area; SFA, subcutaneous fat area; PrFT, perirenal fat thickness; SCCA, subclinical carotid atherosclerosis.

### Cardiometabolic risk factors and cIMT based on PrFT quartiles

**Figure 2:**
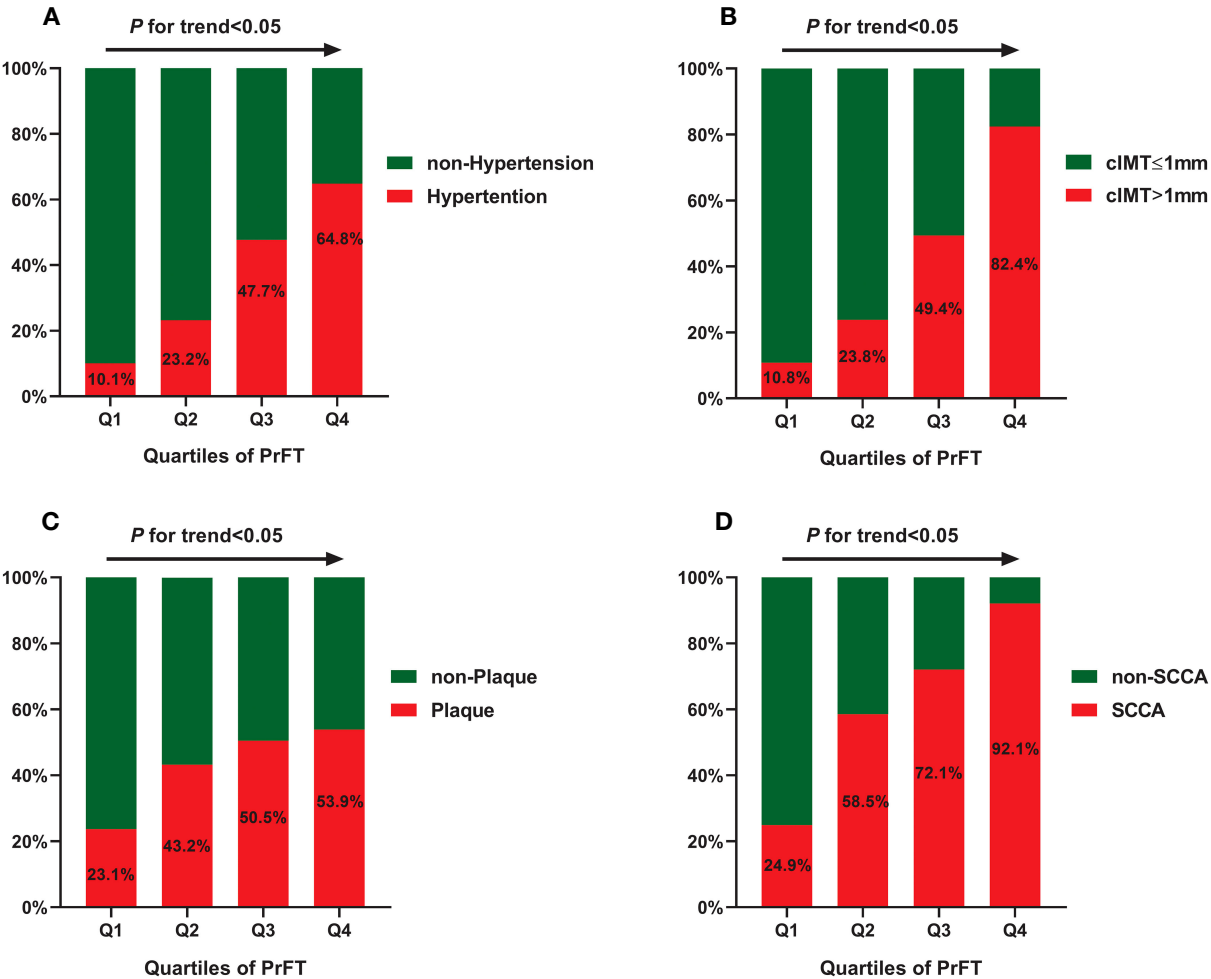
The prevalence of hypertension **(A)**, cIMT > 1 mm **(B)**, plaque **(C)**, and SCCA **(D)** across the PrFT quartiles. cIMT, carotid intima-to-media thickness; PrFT, perirenal fat thickness; SCCA, subclinical carotid atherosclerosis.

**Table 2** presents the cardiometabolic risk factors and cIMT based on the quartiles of PrFT (Q1: <8.60; Q2: 8.60–14.0; Q3: 14.01–16.10; Q4: >16.10). The results revealed significant differences in BMI, WC, SBP, DBP, TG, TC, LDL-c, HDL-c, UA, HOMA-IR, SFA, and VFA among the four quartiles (*p* < 0.05). Meanwhile, increasing trends were observed in the BMI, WC, SBP, DBP, TG, HOMA-IR, SFA, and VFA across the PrFT quartiles. A decreasing trend was also observed in HDL-c across the PrFT quartiles. **Figure 2** illustrates the prevalence of hypertension, cIMT > 1, plaque, and SCCA across the PrFT quartiles. The prevalence of hypertension, cIMT > 1, plaque, and SCCA were 36.4%, 41.5%, 42.8%, and 61.8% in T2DM, respectively. There were significant differences in the prevalence of hypertension, cIMT > 1, plaque, and SCCA among the four quartiles (*p* < 0.05). Furthermore, increasing trends were observed in the prevalence of hypertension, cIMT > 1, plaque, and SCCA across the PrFT quartiles.

**Table 2:** Cardiometabolic risk factors and cIMT based on PrFT quartiles.

Variable	Q1 (*n* = 169)	Q2 (*n* = 164)	Q3 (*n* = 172)	Q4 (*n* = 165)	*p*
Age (years)	52.6 ± 8.9	53.8 ± 7.8	52.9 ± 8.0	53.0 ± 8.2	0.359
BMI (kg/m^2^)	21.6 ± 2.0	23.7 ± 1.7	25.5 ± 1.9	27.2 ± 2.7	<0.001
WC (cm)	79.5 ± 3.4	83.4 ± 3.7	87.9 ± 5.4	92.3 ± 6.7	<0.001
SBP (mmHg)	116.3 ± 10.8	130.1 ± 13.3	142.1 ± 8.5	146.5 ± 18.3	<0.001
DBP (mmHg)	81.7 ± 9.7	83.5 ± 8.6	81.7 ± 9.7	78.2 ± 10.4	<0.001
HbA1c (%)	8.6 ± 0.9	8.7 ± 1.0	8.9 ± 1.4	8.7 ± 0.9	0.141
TG (mmol/L)	1.05 ± 0.72	1.81 ± 0.77	2.24 ± 0.68	3.49 ± 1.58	<0.001
TC (mmol/L)	4.82 ± 1.16	5.25 ± 1.10	5.23 ± 1.30	5.40 ± 1.14	<0.001
HDL-c (mmol/L)	1.33 ± 0.21	1.13 ± 0.19	1.01 ± 0.14	0.94 ± 0.23	<0.001
LDL-c (mmol/L)	3.24 ± 0.92	3.59 ± 0.77	3.72 ± 1.10	3.62 ± 0.94	0.048
UA (μmol/L)	285.5 ± 60.9	339.9 ± 65.9	385.9 ± 72.0	406.8 ± 90.2	<0.001
HOMA-IR	2.89 ± 1.64	4.97 ± 2.37	6.39 ± 2.10	7.95 ± 3.32	<0.001
cIMT (mm)	0.74 ± 0.09	0.93 ± 0.12	1.04 ± 0.10	1.12 ± 0.11	<0.001
VFA (cm^2^)	82.6 ± 13.3	86.7 ± 13.9	91.6 ± 15.8	95.7 ± 17.2	<0.001
SFA (cm^2^)	151.8 ± 21.9	163.3 ± 24.1	173.9 ± 26.7	179.8 ± 23.4	<0.001

BMI, body mass index; WC, waist circumference; SBP, systolic blood pressure; DBP, diastolic blood pressure; HbA1c, glycated hemoglobin A1c; TG, triglyceride; TC, total cholesterol; HDL-c, high-density lipoprotein cholesterol; LDL-c, low-density lipoprotein cholesterol; UA, uric acid; ALT, alanine aminotransferase; HOMA-IR, homeostasis model assessment insulin resistance; VFA, visceral fat area; SFA, subcutaneous fat area; cIMT, carotid intima-to-media thickness; PrFT, perirenal fat thickness; SCCA, subclinical carotid atherosclerosis.

### Correlation of PrFT with cIMT

**Figure 3:**
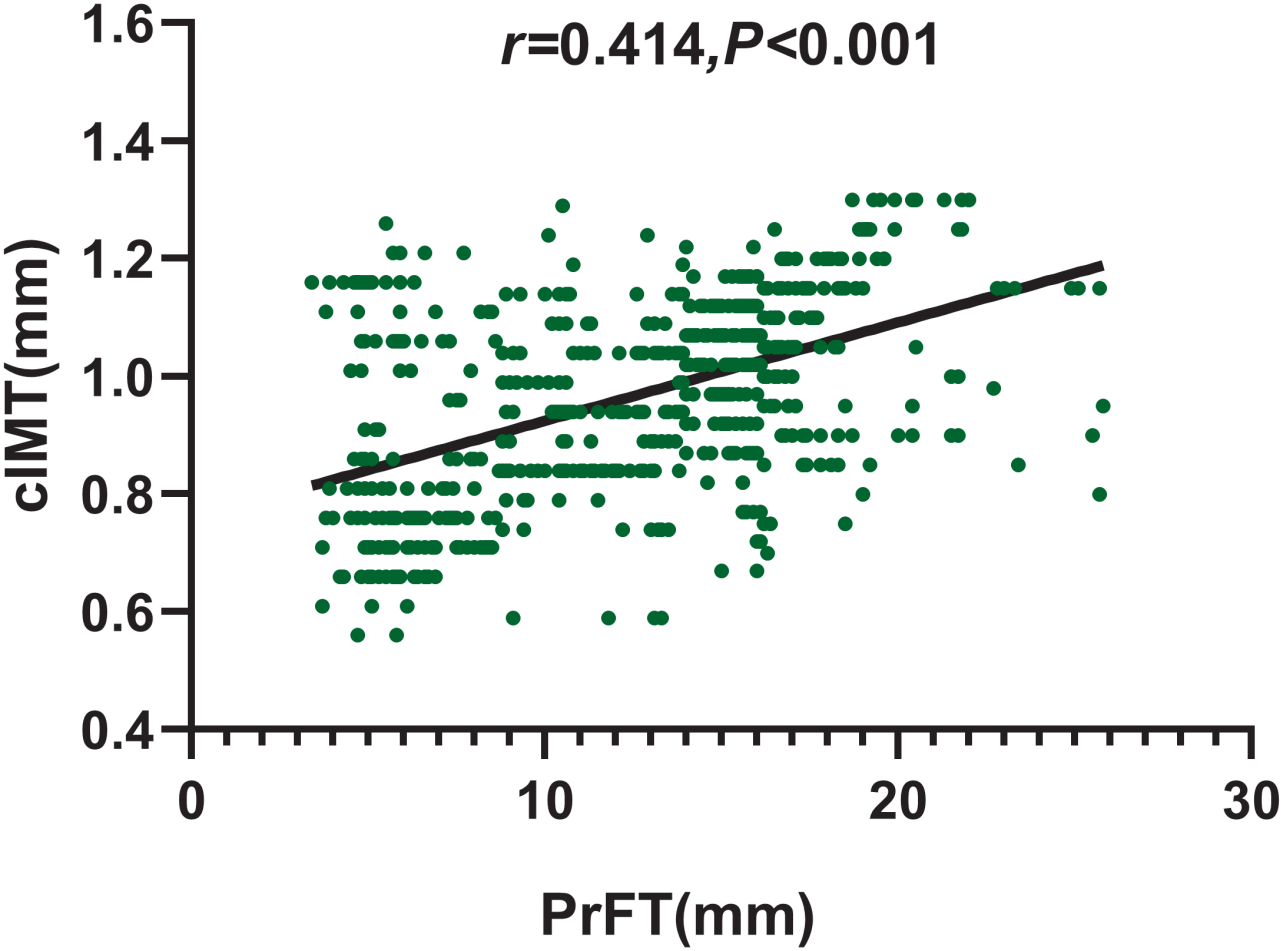
Correlation of PrFT with cIMT in T2DM analyzed by Spearman correlation analysis. cIMT, carotid intima-to-media thickness; PrFT, perirenal fat thickness.

Spearman correlation analysis was conducted to assess the correlation of PrFT with cIMT. As shown in **Figure 3**, PrFT was positively associated with cIMT (*r* = 0.401, *p* < 0.001). Multiple linear regression analysis was conducted to evaluate the correlation between PrFT and cIMT after adjustment for cardiometabolic risk factors. In Model 1, after adjustment for age, sex, BMI, WC, SBP, DBP, diabetic duration, and smoking, PrFT was significantly correlated with cIMT (*β* = 0.317, *p* < 0.001). In Model 2, after adjustment for TG, TC, LDL-c, HDL-c, HbA1c, UA, and HOMA-IR, PrFT was also significantly correlated with cIMT (*β* = 0.235, *p* < 0.001). Furthermore, PrFT maintained a significant correlation with cIMT (*β* = 0.184, *p* < 0.001) after additional adjustment for renal diameters, SFA, and VFA (Model 3).

### Correlation of PrFT with plaque, cIMT > 1 mm, and SCCA

**Figure 4:**
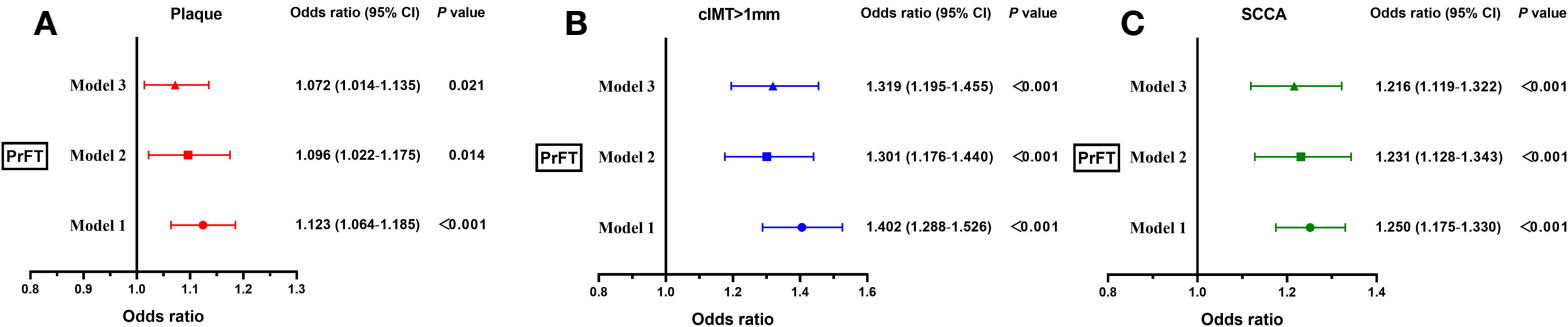
Binomial logistic regression analysis of the association between PrFT and plaque **(A)**, cIMT > 1 mm **(B)**, and SCCA **(C)**. Model 1: adjusted for age, sex, body mass index, waist circumference, systolic blood pressure, diastolic blood pressure, diabetic duration, and smoking. Model 2: additional adjustment for triglycerides, total cholesterol, low-density lipoprotein cholesterol, high-density lipoprotein, uric acid, glycosylated hemoglobin A1c, and homeostasis model assessment insulin resistance. Model 3: further adjustment for renal diameters, and subcutaneous and visceral fat areas. PrFT, perirenal fat thickness; cIMT, carotid intima-to-media thickness; SCCA, subclinical carotid atherosclerosis.

Binomial logistic regression analysis was conducted to evaluate the correlations of PrFT with plaque, cIMT > 1 mm, and SCCA. As illustrated in **Figure 4**, PrFT was independently correlated with plaque, cIMT > 1 mm, and SCCA after adjustment for age, sex, BMI, WC, SBP, DBP, diabetic duration, smoking (Model 1), TG, TC, LDL-c, HDL-c, HbA1c, UA, and HOMA-IR (Model 2). After additional adjustment for renal diameters, SFA, and VFA (Model 3), PrFT remained significantly correlated with plaque, cIMT > 1 mm, and SCCA. The ORs (95% CI) were 1.072 (1.014–1.135), 1.319 (1.195–1.455), and 1.216 (1.119–1.322).

**Figure 5:**
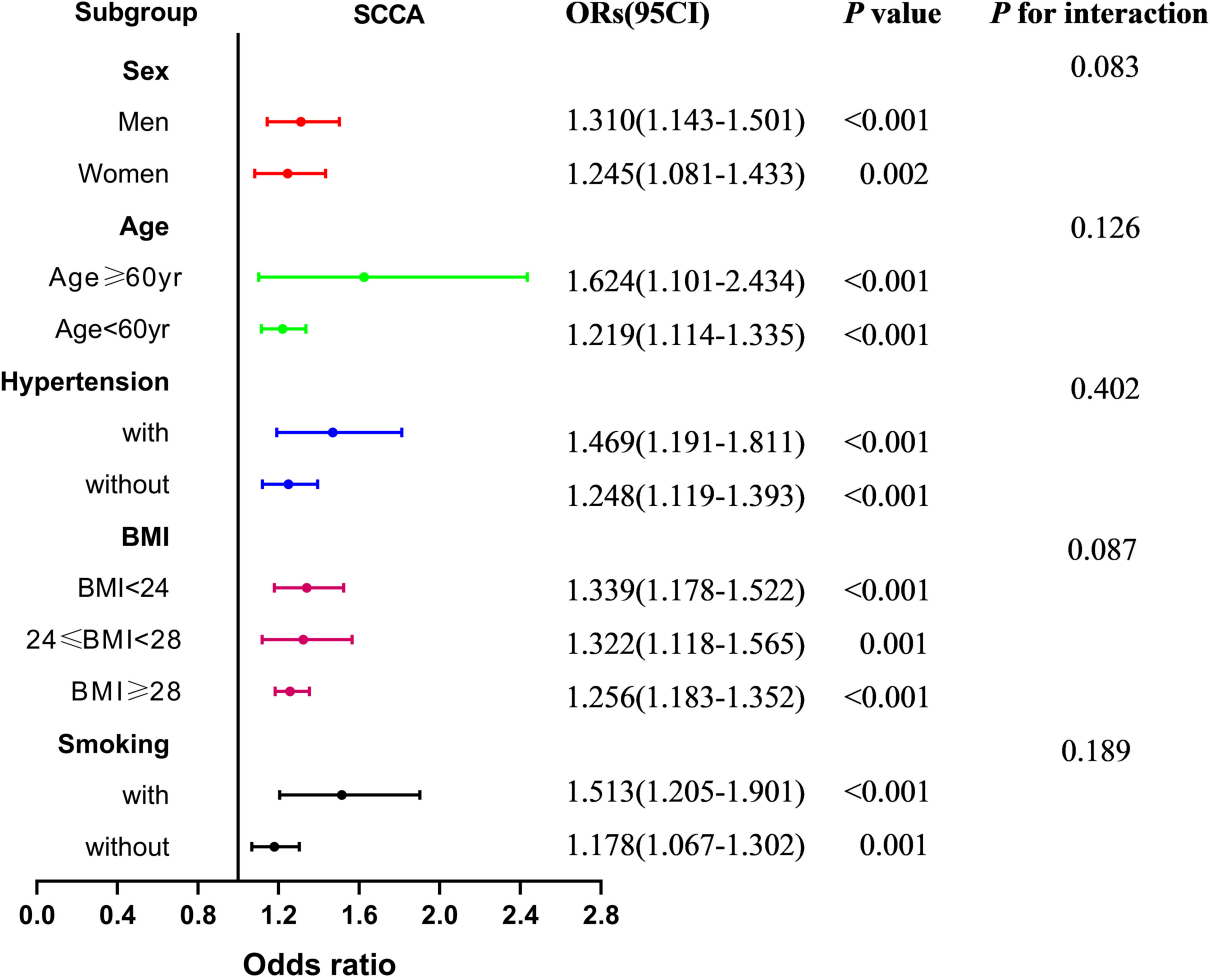
Subgroup analysis of the association between PrFT and SCCA after stratification for age, sex, smoking, hypertension, and body mass index. PrFT, perirenal fat thickness; SCCA, subclinical carotid atherosclerosis.

**Figure 5** presents the subgroup analysis of the association between PrFT and SCCA after stratification for age, sex, smoking, hypertension, and BMI. The results revealed no significant additive interactions between PrFT and SCCA in sex, age, hypertension, smoking, and BMI subgroups (*p* for interaction > 0.05). PrFT was also significantly correlated with SCCA in any subgroups of age, sex, BMI, smoking, and hypertension after Model 3, except for the variables used for stratification.

### Values of PrFT in identifying SCCA

**Figure 6:**
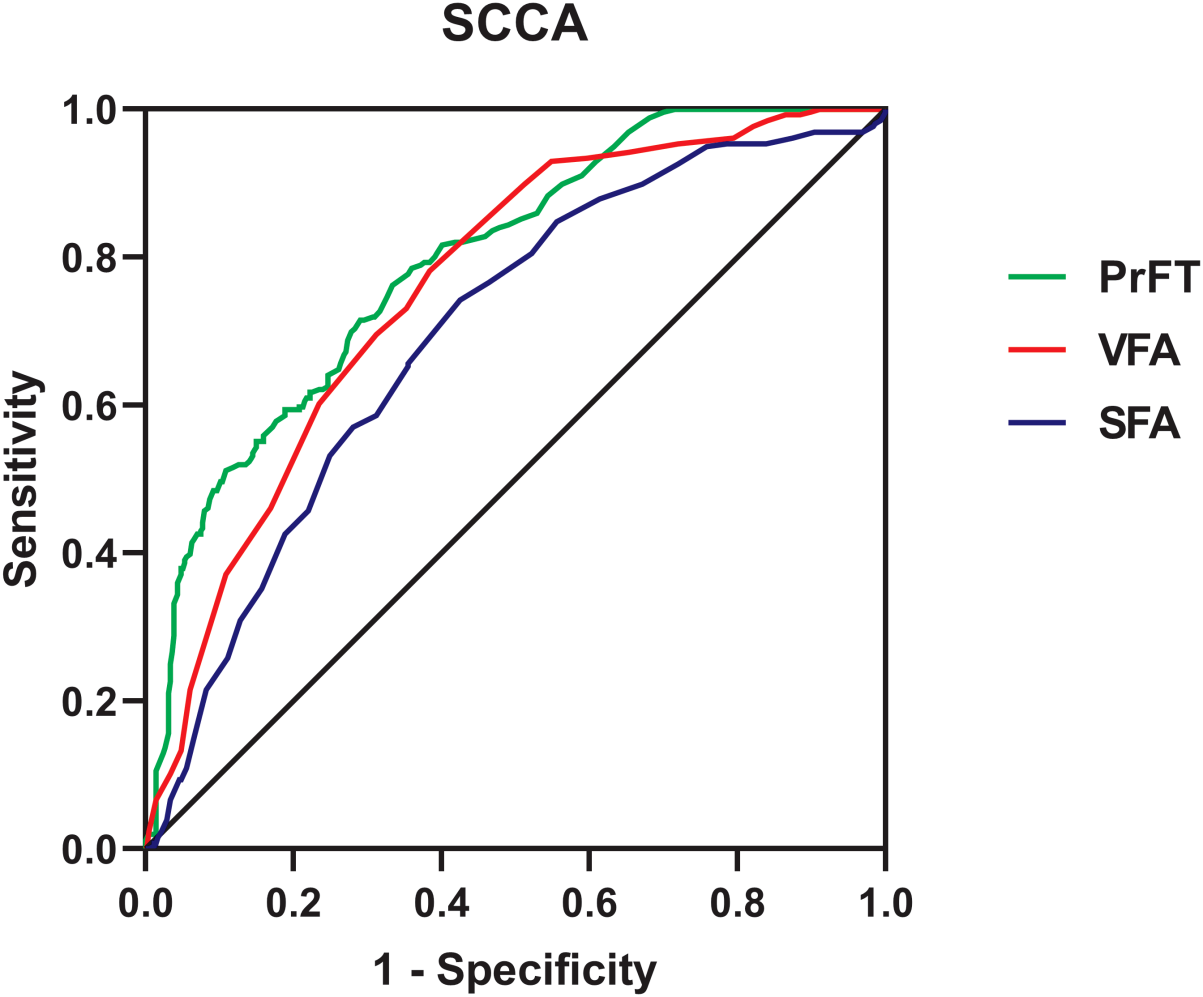
Receiver operating characteristic curves for the cutoff value of PrFT, SFA, and VFA identifying SCCA. PrFT, perirenal fat thickness; SCCA, subclinical carotid atherosclerosis; VFA, visceral fat area; SFA, subcutaneous fat area.

**Figure 6** shows the performance for evaluating the value of PrFT, VFA, and SFA for identifying SCCA. The AUCs (95% CI) of PrFT, VFA, and SFA were 0.794 (0.760–0.828), 0.760 (0.724–0.796), and 0.697 (0.656–0.737), respectively. The AUC of PrFT was significantly higher than VFA (*p* = 0.028) and SFA (*p* < 0.001). The optimal cutoff values of PrFT were 14.0 mm, with a sensitivity of 66.7% and a specificity of 76.2% (**Table 3**).

**Table 3:** ROC curve analysis of PrFT, VFA, and SFA in identifying SCCA.

Variables	AUC (95% CI)	Cutoff value	Sensitivity (%)	Specificity (%)
PrFT (mm)	0.794 (0.760–0.828)	14.0	66.7	76.2
VFA (cm^2^)	0.760 (0.724–0.796)	89.0	61.6	78.1
SFA (cm^2^)	0.697 (0.656–0.737)	168.0	57.5	74.2

PrFT, perirenal fat thickness; VFA, visceral fat area; SFA, subcutaneous fat area; SCCA, subclinical carotid atherosclerosis.

## Discussion

Emerging studies highlighted that PAT might functionally regulate the cardiovascular system and potentially be a target for CVD prevention (12, 23). SCCA was well-recognized as an independent predictor of future CVD events. Work completed on data showed that the association between PrFT and SCCA remained uncertain. Therefore, this study assessed the association between PrFT and SCCA. Meanwhile, we further compared the value of PrFT and VFA in identifying SCCA. The results revealed that PrFT was significantly increased in participants with SCCA. Meanwhile, PrFT was independently correlated with cIMT, plaque, cIMT > 1 mm, and SCCA after adjustment for cardiometabolic risk factors. Moreover, PrFT remained significantly associated with SCCA in subgroup analysis of age, sex, BMI, smoking, and hypertension. Furthermore, PrFT was superior to VFA and SFA in identifying SCCA.

T2DM is closely associated with SCCA. The prevalence of SCCA was relatively high (61.8%) in our study. Obesity involves excessive accumulation of adipose tissue, which is thought to be a leading risk factor for CVD and T2DM. VAT is most important in adipose biology categories based on fat depots’ anatomical and physiological characteristics. VAT is active in releasing bio-active factors such as leptin, adiponectin, tumor necrosis factor-α, interleukin-6, interleukin-8, and MCP-1 that are involved in the pathogenesis of CVD, metabolic disorders, and T2DM (24, 25). Recent studies have shown that the CVD risk associated with obesity is particularly relevant for VAT. Increased accumulation of VAT not only drives the increased prevalence of T2DM and SCCA but also accelerates the development of SCCA in T2DM (26). Atherosclerosis was previously considered a lipid-storage disease. In comparison, emerging evidence has demonstrated that it was a subacute inflammatory condition of the vessel wall characterized by infiltration of macrophages and T cells. Metabolic disorders can trigger chronic low-grade inflammation at almost every step of the atherogenic process, which plays a major role in developing atherosclerosis (27, 28). Hence, we can observe that the cardiometabolic risk factors like BMI, WC, SBP, DBP, TC, LDL-c, UA, HOMA-IR, SFA, and VFA were significantly increased in the SCCA group. Hypertension, insulin resistance, and dyslipidemia are the main cardiometabolic risk factors that can accelerate the development of atherosclerosis. Previous studies suggested that excessive PAT increases the risk of hypertension, insulin resistance, and dyslipidemia. De Pergola et al. observed that increased PrFT was positively associated with increased mean 24-h BP levels in overweight and obese subjects (29). Maria et al. also found that decreased anti-hypertensive medications and SBP levels were significantly associated with decreased PrFT in hypertensive obese subjects after sleeve-gastrectomy surgery (30). Meanwhile, increased PrFT was also associated with reduced HDL-c and increased insulin resistance in overweight and obese subjects (31). The results of our study were consistent with the previous studies. Increasing trends were observed in the SBP, DBP, TG, and HOMA-IR across the PrFT quartiles. A decreasing trend was also observed in HDL-c.

Increased cIMT and plaque are validated markers of atherosclerosis. Previous studies found that increased PAT was associated with increased cIMT. Bassols et al. observed that PAT was independently associated with cIMT after adjusting for BMI, gender, age, and metabolic parameters in 142 overweight (*β* = 0.250) and 142 obese (*β* = 0.254) children (16). Okeahialam et al. found that PrFT measured by ultrasound was positively correlated with cIMT (*β* = 0.195) in 221 overweight or obese subjects (32). Consistent with the above studies, our studies revealed that PrFT was independently associated with cIMT (*β* = 0.184) after adjustment for cardiometabolic risk factors, SFA, and VFA in 670 participants with T2DM. The correlation of SCCA with VAT volume and EFT has been evaluated in previous studies. Several clinical studies observed that VAT volume and EFT were independent predictors of SCCA and plaque (10, 11, 33–35). As VAT volume and EFT, this study also found that PrFT was significantly correlated with plaque, cIMT > 1 mm, and SCCA independent of cardiometabolic risk factors, SFA, and VFA. Moreover, PrFT remained correlated considerably with SCCA in subgroup analysis after stratification for age, sex, smoking, hypertension, and BMI. Ohashi et al. found that VFA was significantly associated with the presence and extent of CAC as a marker of subclinical atherosclerosis in Japanese patients (18). As expected, our study also revealed that PrFT had a good identifying value for SCCA. Furthermore, the AUC of PrFT was significantly higher than VFA and SFA. These findings indicated that PrFT might be a better marker of SCCA in T2DM.

Among the underlying mechanisms that attempt to explain the association between PrFT and SCCA, PAT’s unique structure and biology may play critical roles. PAT shares the same developmental origin as typical VAT and thus has the same roles as VAT in cardiovascular and metabolism systems. However, there are some differences between typical VAT and PAT in histology, physiology, and functions. PAT has a complete blood supply system, lymph fluid drainage, and innervation, making it more like an organ than connective tissue (14). Furthermore, the unique morphological basis ensures that perirenal fat plays more functional roles in energy metabolism and adipokine bio-transformation than typical VAT (36). Intracellular lipid accumulation by foam cells, vascular smooth muscle cells, and T lymphocytes characterize the atherosclerotic lesion. This can increase the intima-media thickness and progress to atherosclerotic lesions due to chronic endothelium injury. The chronic inflammatory response was considered the critical factor of atherosclerosis and started from the earliest stages of pathology initiation. Emerging evidence highlighted that perirenal fat could regulate the cardiovascular system via neural reflexes, adipokine secretion, adipocyte interactions, and paracrine substances (37, 38). PAT is highly active in cytokine synthesis by local immune cells like T lymphocytes and monocytes/macrophages (39), which may play essential roles in triggering the inflammatory response in the endothelium. In addition, increasing evidence demonstrated that inflammatory factors like tumor necrosis factor-α, interleukin-6, and interleukin-8 can also be released from PAT, which is involved in the pathogenesis of atherosclerosis (40).

### Strength and limitation

This study adjusted several potential confounding variables and included enough population to evaluate the association between PrFT and SCCA. Several limitations need to be mentioned in this study. First, this study was designed as a cross-sectional study based on prospectively collected data from a single center in the Chinese population without follow-up. Second, the PrFT was measured by CT. The radiation may limit its use in some conditions, such as pregnancy. Third, the measurement of PrFT may vary under different detection methods. The optimal cutoff values of PrFT for SCCA may not apply to participants who underwent ultrasound examinations.

## Conclusion

It is well-acknowledged that participants can benefit from intensive primary prevention strategies at an earlier stage of atherosclerosis. Our study found a novel risk factor and promising target for SCCA. PrFT was independently associated with cIMT, plaque, cIMT > 1 mm, and SCCA and had a good identifying value for SCCA than VFA and SFA. These findings indicated that PrFT might be a superior obesity-related marker of SCCA in T2DM. More attention should be paid on T2DM with excessive accumulation of PAT.

## Data availability statement

The original contributions presented in the study are included in the article/**Supplementary Material**. Further inquiries can be directed to the corresponding author.

## Ethics statement

The studies involving humans were approved by Ethical Committee of Longyan First Affiliated Hospital of Fujian Medical University. The studies were conducted in accordance with the local legislation and institutional requirements. The participants provided their written informed consent to participate in this study.

## Author contributions

XG: Data curation, Formal Analysis, Investigation, Methodology, Software, Writing – original draft. JW: Data curation, Investigation, Methodology, Software, Writing – original draft. MT: Data curation, Investigation, Writing – original draft. WW: Data curation, Investigation, Methodology, Writing – review & editing, Formal Analysis, Writing – original draft.

## Supplementary material

Supplementary Figure 1 Perirenal adipose tissue was differentiated from other tissues at the renal venous plane (*) by density. The average maximal distance (blue arrow line) between the kidney’s posterior wall and the abdominal wall’s inner limit on the left and right side was measured as perirenal fat thickness.
