# Supplementary figures and images for: Perirenal fat thickness as a superior obesity-related marker of subclinical carotid atherosclerosis in type 2 diabetes mellitus
Source: Front Endocrinol (Lausanne). 2023 Oct 27;14:1276789. doi: 10.3389/fendo.2023.1276789 (PMC10641744; doi:10.3389/fendo.2023.1276789)

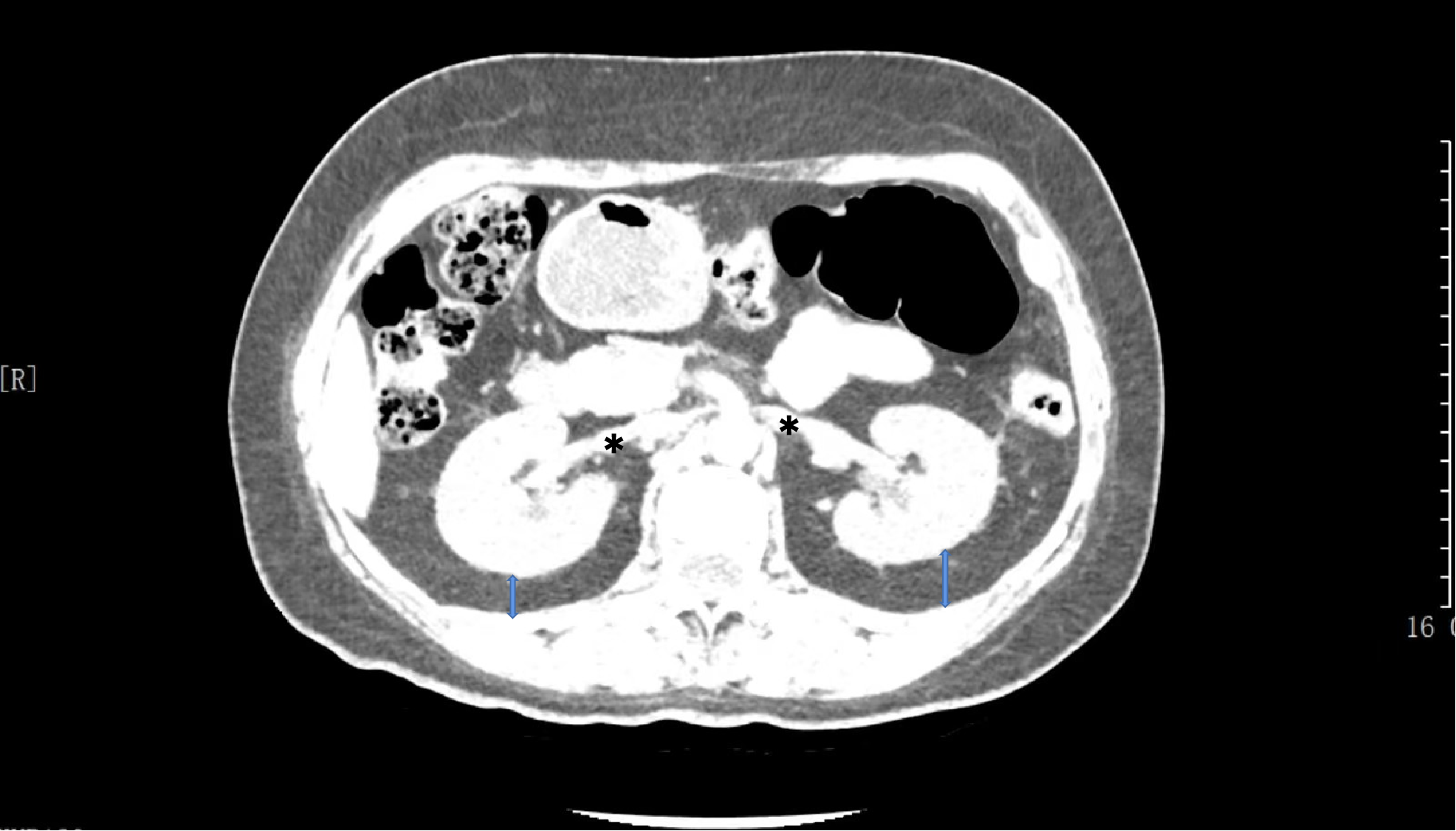

Supplement: Supplementary Figure 1 — Perirenal adipose tissue was differentiated from other tissues at the renal venous plane (*) by density. The average maximal distance (blue arrow line) between the kidney’s posterior wall and the abdominal wall’s inner limit on the left and right side was measured as perirenal fat thickness. [file Image_1.tif]
